# Proximate Composition and Fatty Acid Profile of Gilthead Seabream (*Sparus aurata*) Fed with *Pelvetia canaliculata*-Supplemented Diets: An Insight towards the Valorization of Seaweed Biomass

**DOI:** 10.3390/foods12091810

**Published:** 2023-04-27

**Authors:** Madalena Antunes, Marta Neves, Damiana Pires, Ricardo Passos, Beatriz do Carmo, Carolina F. Tchobanov, Sara Forte, Mariana Vaz, Teresa Baptista, Carla Tecelão

**Affiliations:** 1 MARE—Marine and Environmental Sciences Centre, ARNET—Aquatic Research Network, ESTM, Politécnico de Leiria, 2520-630 Peniche, Portugal; 2 School of Tourism and Maritime Technology, Politécnico de Leiria, 2520-641 Peniche, Portugal

**Keywords:** aquaculture feed, fatty acid profile, health lipid indices, *Pelvetia canaliculata*, proximate composition, *Sparus aurata*

## Abstract

Seaweeds are a sustainable source of protein and lipids that may be used to replace fish by-products in aquaculture feed. This study aimed at using the macroalgae *Pelvetia canaliculata* as an ingredient in gilthead seabream (*Sparus aurata*) feed, either as freeze-dried powder or as algae residue (waste) that was obtained after the supplementation of sunflower oil. The formulated diets and the fish muscle were analyzed concerning the proximate composition and the fatty acid profile. The health lipid indices hypocholesterolemic/hypercholesterolemic (h/H), atherogenic (AI), thrombogenic (TI), as well as n-3/n-6 and polyunsaturated fatty acid/saturated fatty acid (PUFA/SFA) ratios were calculated. Additionally, the peroxidizability index (PI) was determined. No differences were observed in the proximate composition of fish muscle regardless of the diet used. Fish fed a diet supplemented with 10% of algae waste (W10) stand out for the highest content in oleic acid (C18:1 n-9), and the lowest in both linoleic (C18:2 n-6) and palmitic (C16:0) fatty acids. All fish samples showed values of health lipid indices within the limits recommend for a nutritional balanced diet. These results highlight that fish fed diets supplemented with *P. canaliculata* are sources of healthy lipids that might be consumed on a regular basis to prevent cardiovascular diseases.

## 1. Introduction

Food production, namely of animal origin, has been following the increase in world population. Therefore, the search for available, less expensive, and more sustainable food sources, following a circular economy concept, has been a major concern for the scientific community [1,2,3].

The sustainability of the aquaculture sector requires the implementation of cost-effective strategies to increase production without compromising the nutritional quality of farmed fish, which is highly dependent on diet composition [4,5,6]. A viable approach is to reduce feed costs, which may represent up to 50% of the production budget in an intensive regime, by using less expensive ingredients in diet formulations. In this sense, seaweeds stand out as a promising and sustainable source of nutrients to supplement aquaculture feeds [7,8,9].

Seaweeds are photosynthetic organisms that do not compete for arable land, do not require fresh water, and contribute to reducing the atmospheric CO_2_ through photosynthesis [10]. Their nutritional composition is highly dependent on species characteristics and environmental conditions [11]. Nevertheless, even though the nutritional properties of these organisms have not been studied as much as those of vascular plants, it is well stated that their chemical composition may have high contents of vitamins, minerals, and non-starch polysaccharides, moderate amounts of protein (10 to 30 g/100 g DW), and low lipid contents (0.3 to 7.2 g/100 g DW). The lipid fraction is usually rich in mono- and polyunsaturated fatty acids (PUFA), such as eicosapentaenoic acid (EPA) and docosahexaenoic acid (DHA) [11]. Seaweeds are also known to be a reliable source of bioactive compounds with interesting properties, such as antioxidant, antimicrobial, antiviral, anti-inflammatory, and antitumoral [12,13]. Among them, species that inhabit the intertidal zone are worth of attention for the synthesis of compounds with bioactive properties that may grant them protection against environmental variations, namely the alternation of emersion/immersion phases and day/night cycles. Brown seaweeds, mainly belonging to the order Fucales, such as *Pelvetia canaliculata*, may accumulate high amounts of secondary metabolites, namely carotenoids and phenolic compounds that have a protect effect against exposure to UV radiation [14].

Several studies have shown the benefits of using macroalgae as ingredients for aquaculture feed on the growth, stress resistance, immune system, and nutritional quality of fish [15,16,17]. However, depending on the species, high concentrations of seaweeds in diets may have toxic effects on farmed fish [11,18,19,20].

*Sparus aurata* is one of the most relevant aquaculture species in the Mediterranean and European Union. The large-scale production of this species started in the 1980s and has shown robust growth in recent years [21]. Several studies have reported the effects of using macroalgae (such as *Laminaria* [7], *Gracilaria* [19,20,22], *Ulva* [16,20,22], and *Pterocladia* [23]) in *Sparus aurata* feed.

This study proposes a different approach for macroalgae use in *Sparus aurata* aquaculture feed. A previous work, carried out by our research group, reported the use of the seaweed *Pelvetia canaliculata* in sunflower oil supplementation aiming to increase its oxidative stability [24]. This procedure led to the production of algal residue that still has interesting nutritional value. In this sense, the present study proposes a strategy to valorize this biomass. Farmed *Sparus aurata* juveniles were fed with diets supplemented with *P. canaliculata*, either as freeze-dried powder or as waste, i.e., residue left after sunflower oil enrichment. The diets and fish muscle were characterized based on their proximate compositions and fatty acid profiles to sustain the viability of this approach for aquaculture application.

## 2. Materials and Methods

### 2.1. Ethics Statement

The current study was carried out according to the guidelines on the protection of animals used for scientific purposes from the European Directive 2010/63/EU and under the project authorization 0421/000/000/2019.

### 2.2. Chemicals and Biological Materials

The brown macroalga *Pelvetia canaliculata* L. was harvested at the beach of Pedras do Corgo, Portugal (41°14′55.52″ N, 8°43′29.89″ W), in April 2021. Gilthead seabream (*Sparus aurata*) juveniles were acquired from EPPO—Aquaculture Research Station (Portuguese Institute for Sea and Atmosphere). Kjeldahl catalyst tablets were purchased from VWR (Radnor, Pennsylvania, EUA), analytical standards Supelco 37 Component FAME Mix, C17 FAME; PUFA 1 and PUFA 3 were acquired from Sigma-Aldrich (St. Louis, MO, USA). The remaining reagents were of analytical grade and obtained from various sources.

### 2.3. Experimental Design and Diets Formulation

**Figure 1:**
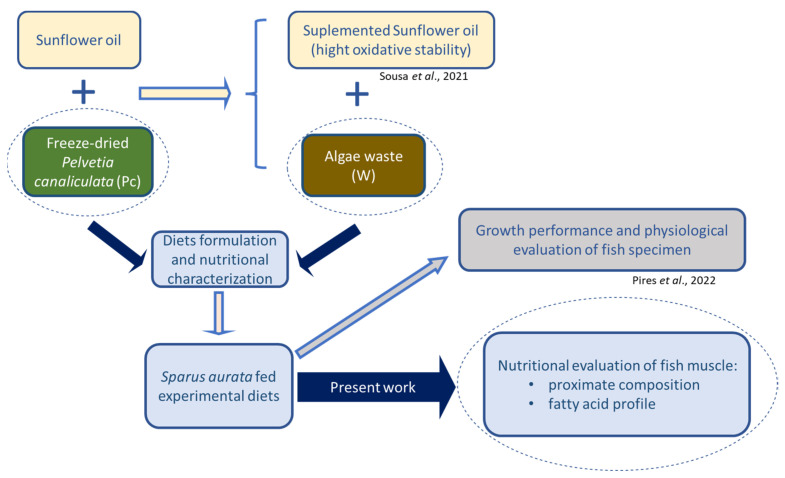
Integrative perspective of the work developed in the present study [24,25].

*P. canaliculata* was cleaned from extraneous matter, frozen at −80 °C, freeze-dried (Telstar, Lyoquest-85, Telstar Portugal, Lisbon, Portugal), ground to powder, and stored at room temperature, protected from light, until use. The alga was incorporated in diet formulations either as powder or as waste that was obtained after sunflower oil supplementation, as described by Sousa et al. [24]. Briefly, 12.5% (*m/v*) of freeze-dried *P. canaliculata* was added to sunflower oil and submitted to ultrasound-assisted extraction for 20 min. After, the mixture was filtered, and the solid residue (remaining algal biomass) was used as an ingredient for aquaculture feeds. A specialized company (SPAROS, Olhão, Portugal) formulated six isoproteic and isolipidic diets, using soybean oil in different proportions to adjust the fat content in the feeds. *P. canaliculata* was used either as freeze-dried powder, in concentrations of 1% (Pc1), 5% (Pc5), and 10% (Pc10), or as waste, in concentrations of 1% (W1) and 10% (W10). Additionally, a control diet (CT) without *P. canaliculata* was prepared. The description of all ingredients used in diet formulations was previously reported by Pires et al. [25]. Aiming at showing the overall work process in an integrative perspective, the experimental design is presented in Figure 1.

### 2.4. Rearing Conditions

The entire trial lasted for 44 days and was carried out at the Aquaculture Laboratory of MARE—Polytechnic of Leiria (Peniche, Portugal), as described by Pires et al. [25]. After two weeks of quarantine, the juveniles of gilthead seabream (*Sparus aurata*) were individually weighed (15.41 ± 3.69 g) and randomly distributed through eighteen 60 L aquaria, reaching a stock density of 5.14 ± 0.26 kg m^−3^, with 20 individuals per aquarium. The experimental setup was acclimated for one week prior to the start of the assay. The aquaria were divided into 6 closed water recirculation systems (RAS), 3 for each diet. During the entire trial, the mortality, water temperature (20.49 ± 1.07 °C), salinity (32.79 ± 0.35), pH (8.07 ± 0.19), and dissolved oxygen (91.87 ± 3.36%) were recorded daily. Fish were hand-fed, ad libitum, three times a day. They were fasted 24 h before sampling and then sacrificed. The nutritional analyses were obtained from two pool samples from each aquarium corresponding to fish fillets from three individuals (*n* = 6). 

### 2.5. Proximate Composition

Experimental diets were ground to powder and stored at room temperature, protected from light and moisture, until use. Fish muscle samples were weighed, frozen at −80 °C, freeze-dried, ground to powder, and stored at the same conditions as experimental diets.

#### 2.5.1. Moisture Content

Experimental diets were analyzed for moisture content according to AOAC (930.15, 2016) [26], with slight modifications. The samples (1 g) were weighed into a porcelain crucible, previously dried (5 h at 200 °C), and heated at 105 °C in a ventilated oven (Binder, Tuttlingen, Germany), until constant weight was obtained. Fish muscle moisture was determined gravimetrically, by calculating the water loss after freeze-drying. Results were represented as percentage of fresh weight (% FW). 

#### 2.5.2. Ash Content

Ash content of either diets or fish samples was determined according to AOAC (942.05, 2016) [26]. Each sample (1 g) was weighed into a porcelain crucible, previously dried (5 h at 200 °C), and incinerated in a muffle (Nabertherm, Liliemthal/Bermen, Germany) at 500 °C for 12 h. Results were expressed as % FW.

#### 2.5.3. Protein Content

The protein contents of the experimental diets and of fish muscle were estimated by the Kjedahl method, following AOAC (940.25, 2016) [26], using a conversion factor of 6.25. Briefly, the samples (250 mg or 150 mg of diets and muscle, respectively) were digested (Digestor 2006; Foss, Denmark) with a catalyst tablet and 25 mL of H_2_SO_4_ (97%) at 220 °C for 30 min and 400 °C for 90 min. Deionized water (70 mL) was added to cold digested samples that were distillated (Kjeltec 2100, Foss, Denmark) with 100 mL of NaOH (40% *m/v*). The distillate was collected in 30 mL of H_3_BO_3_ (4% *m/v*) with methyl red and bromocresol green as pH indicators for further titration with standardized HCl 0.1 M. Blank assay was prepared at the same conditions as samples. The results were calculated according to Equation (1) and expressed as % FW:

(1) $$\mathrm{Protein}\;(\%\;\mathrm{FW})=\frac{\left[\mathrm{HCl}\right]\times (V_{S}-V_{B})\times 14\times 6.25}{m}$$

where [HCl]—chloride acid concentration (M); V_s_ and V_B_—volume of HCl spent for samples and blank titration, respectively (mL); m—sample mass (mg).

#### 2.5.4. Total Lipid Content

Total lipid content was determined according to Folch et al. [27], with some modifications as described by Neves et al. [28]. Diets (1 g) or fish muscle (0.75 g) were mixed with 1 mL of deionized water and 10 mL of Folch reagent, under vortex stirring for 5 min. After the addition of 1.2 mL of NaCl (0.8%), the mixtures were vortexed for a further 2 min and then centrifuged (5 min at 6000 rpm) for phase separation. The lower phase was collected to a round-bottom flask after filtration through an anhydrous sodium sulphate column. The same extraction procedure was repeated with 5 mL of chloroform. Finally, the organic phase was evaporated in a rotary evaporator (Heidolph 2, LAB1ST, Shanghai China) and the lipid residue was dried at 60 °C until constant weight. The lipid content was expressed as % FW.

### 2.6. Fatty Acid Profile

The fatty acid (FA) profiles of freeze-dried *Pelvetia canaliculta*, diets, and fish muscle were determined according to Fernández et al. [29]. Samples (50 mg) were mixed with 2 mL of methanolic sulfuric acid (H_2_SO_4_) solution 2% (*v/v*) and heated at 80 °C for 2 h. After cooling, the obtained fatty acid methyl esters (FAME) were extracted with *n*-hexane (2 mL) and analyzed by gas chromatography (GC) following the conditions described by Neves et al. [28]. Fatty acids were identified by comparison of their retention times with those of Supelco 37, PUFA 1, and PUFA 3 standard mixtures. The results were expressed as percentage of total fatty acids (% total FA).

### 2.7. Health Lipid Indices Calculation

Hypocholesterolemic/hypercholesterolemic (*h*/*H*), atherogenic (*AI*), thrombogenic (*TI*), and peroxidizability (*PI*) indices were calculated based on the fatty acid profile of *Sparus aurata* muscle, according to the following Equations (2)–(5) [30]:

(2) $$h/H=\frac{\begin{array}{c} C18:1n9+C18:1n7+C18:2n6+C18:3n6+C18:3n3+C20:3n6+\\ C20:4n6+C20:5n3+C22:4n6+C22:5n3+C22:6n3 \end{array}}{C14:0+C16:0}$$

(3) $$AI=\frac{C12:0+4\times \left(C14:0\right)+C16:0}{MUFA+n3+n6}$$

(4) $$TI=\frac{C14:0+C16:0+C18:0}{0.5\times MUFA+3\times n3+0.5\times n6+n3/n6}$$

(5) $$\begin{array}{ll} PI= & 0.025\times (monoenoic\;acids)+dienoic\;acids+2\times (trienoic\;acids)\\ & +4\times (tetraenoic\;acids)+6\times (pentaenoic\;acids)\\ & +8\times (hexaenoic\;acids) \end{array}$$

### 2.8. Statistical Analyses

Statistical analyses were performed with the SPSS software (v27, IBM, Armonk, New York, NY, USA) and significance level set at *p* ≤ 0.05. The data were analyzed by analysis of variance (one-way ANOVA) followed by Tukey’s post hoc test. The assumptions of ANOVA were verified through the Kolmogorov–Smirnov normality test and Levenne homogeneity. If the assumptions were not observed, Games–Howell nonparametric test was applied. All the presented values correspond to mean ± standard deviation of three independent samples. 

A principal component analysis (PCA) was performed using CANOCO 4.5 software to identify the main variation in the fatty acid profile of muscle of gilthead seabream fed with the different diets. PCA was applied to the log-transformed data set of all analyses.

## 3. Results and Discussion

The experimental diets supplemented with *P. canaliculata*, either as powder or as waste, were characterized based on their proximate compositions and fatty acid (FA) profiles. The influence of these diets on the nutritional value of gilthead seabream fillets were evaluated. 

### 3.1. Nutritional Characterization of Algal Biomass

In a previous study carried out by our research group, the proximate composition of algal biomass was studied, regarding both the freeze-dried *Pelvetia canaliculata* and the waste left after sunflower oil supplementation [25]. Freeze-dried powder was mainly composed of carbohydrates (*ca.* 76%), ash (about 21%), and lower amounts of protein (*ca.* 8%) and lipids (*ca.* 5%). The waste had 45% carbohydrates, 35% lipids, 15% ash, and 5% protein. The higher lipid content in waste results from oil adsorption in the biomass recovered from sunflower oil. Aiming at reaching a complete nutritional characterization of *P. canaliculata*, its fatty acid profile was analyzed in this study (Table 1). Polyunsaturated fatty acids (PUFA) were the major class, mainly comprising n-6 FA (C20:4 and C18:2), followed by monounsaturated fatty acids (MUFA), mostly C18:1 n-9 and C16:1 n-7, and saturated fatty acids (SFA), C16:0, C14:0, and C18:0. α-Linolenic acid (ALA) and Eicosapentaenoic acid (EPA) were the most abundant n-3 PUFA, accounting for 10% of total FA. These values are in agreement with those reported by Schmid et al. [31] and Maehre et al. [32].

### 3.2. Nutritional Characterization of Experimental Diets

**Figure 2:**
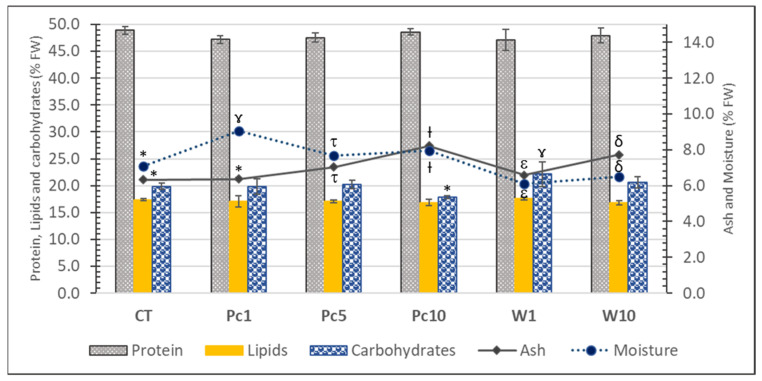
Proximate composition (% FW) of the experimental diets: CT—control diet; Pc1—diet supplemented with 1% freeze-dried *P. canaliculata*; Pc5—diet supplemented with 5% freeze-dried *P. canaliculata*; Pc10—diet supplemented with 10% freeze-dried *P. canaliculata*; W1—diet supplemented with 1% *P. canaliculata* waste; W10—diet supplemented with 10% *P. canaliculata* waste. For each parameter, a distinct symbol (asterisc and gamma symbols) means significative differences (*p* < 0.05) between samples.

The proximate composition of experimental diets was described by Pires et al. [25] and is summarized in Figure 2. Protein stands out as the major nutrient (*ca.* 48%), followed by carbohydrates (ranging from 17.85 ± 0.30 to 22.1 ± 2.3%), lipids (*ca.* 17%), moisture (6.12 ± 0.05 to 9.08 ± 0.11%), and ash (6.33 ± 0.07 to 8.22 ± 0.04%). No statistically significant differences in the protein and lipid contents were observed, which confirms the initial request for isolipidic and isoproteic diets. Algal biomass increased the ash content in all supplemented diets in comparison with the control, reflecting seaweed’s richness in minerals, namely Mn, I, Na, K, or Zn [33]. Concerning carbohydrate content, the W1 and Pc10 diets had the highest and the lowest values, respectively. However, none of the supplemented diets showed statistically significant differences in comparison with the control.

The FA profile of the experimental diets is shown in Table 2. The most abundant FAs in all diets were linoleic (C18:2 n-6), oleic (C18:1 n-9), and palmitic (C16:0). These FAs are also the most abundant in the solid residue (remaining algal biomass) left after sunflower oil supplementation, previously named as waste, and in the soybean oil, used as a fat source in feed formulation [34,35]. The highest content in MUFA (mainly C18:1 n-9) and the lowest content in PUFA (mainly C18:2 n-6) was observed in the W10 diet. These might be due to different proportions of soybean oil/sunflower oil in the experimental diets. In fact, the W10 diet contains only 2.6% of soybean oil, rather below the amounts added to the remaining diets (5.6 to 6.0%) [25]. Additionally, the W10 formulation stands out for the lowest SFA content, especially due to the lower amount of C16:0. The W10 diet also has the highest ratio n-3/n-6, which may be an advantage from a nutritional point of view. Concerning the content of essential FAs, it was observed that the addition of algae waste (W1 and W10) led to a decrease in ALA (C18:3 n-3) in comparison with the remaining diets. Conversely, the amount of docosahexaenoic acid (DHA, C22:6 n-3) and EPA (C20:5 n-3) was always lower in diets containing algal biomass in comparison with the control. 

### 3.3. Nutritional Composition and Fatty Acid Profile of S. aurata Muscle

**Figure 3:**
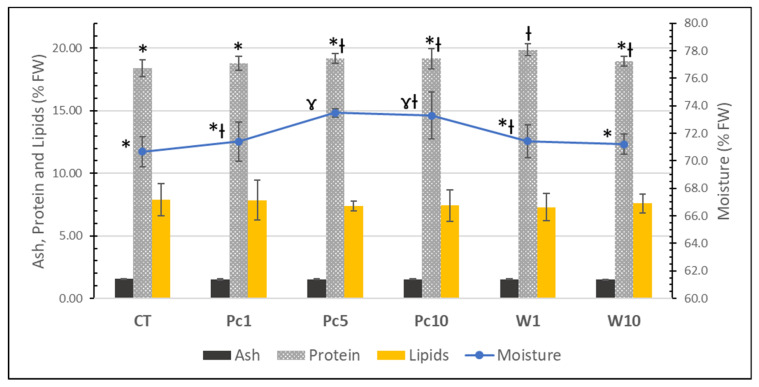
Proximate compositions of the muscle of *S. aurata* fed with the formulated diets: CT, Pc1, Pc5, Pc10, W1, and W10. For each parameter, a distinct symbol (asterisc and gamma symbols) means significative differences (*p* < 0.05) between samples.

During the 44-day trial, fish fed different diets showed a similar performance concerning mortality, growth rate, feed uptake, and somatic indices, as reported by Pires et al. [25]. Taking into consideration that the *S aurata* produced in aquaculture is for human consumption, it is of huge importance to compare its nutritional profile to assess the viability of using the algal biomass in diets. Therefore, the proximate compositions and fatty acid profiles of fish fed experimental diets were analyzed. The proximate compositions of fish muscle (fillets) are shown in Figure 3. No relevant differences were observed in the nutritional composition of fish samples, regardless of the diet tested. As expected, fillets are mainly composed of water (ranging from 70.7 ± 1.1 to 73.5 ± 0.3%), followed by protein (18.4 ± 0.7 to 19.9 ± 0.5%), lipids (7.3 ± 1.1 to 7.9 ± 1.6%), and lower amounts of ash (1.51 ± 0.02 to 1.55 ± 0.03%). These results are in agreement with those attained by Martínez-Llorens et al. [36], that reported no differences in the proximate composition of fish muscle when fish oil was replaced by soybean oil in gilthead seabream fingerling diets. The study carried out by Mechlaoui et al. [37] also showed that diets with selenium, either in organic or inorganic forms, had no influence on the nutritional profile of gilthead seabream muscle. Similar values of protein (ca. 19%) and ash (ca. 1.5%) were obtained in comparison with the present study. However, lower lipid contents (ca. 4–5%) and higher moisture (ca. 73–77%) were reported. 

Concerning the FA profile of *S. aurata* (Table 3), the muscle of fish fed W10 stands out for the highest content of C18:1 n-9 and the lowest of both C18:2 n-6 and C16:0, reflecting feed intake. No statistically significant differences were observed for these major FAs in the remaining fish samples, in line with diet composition. Martínez-Llorens et al. [36] also described a correlation between the FA profile of gilthead seabream muscle and the feed intake, observing an increase in C18:2 n-6 with higher proportions of soybean oil in fish diets. Regarding n-3 FA, fish fed Pc5 and Pc10 showed the highest content of DHA (ca. 6.4%), whereas no differences were observed for EPA in all samples, ranging from 5.25 ± 0.21 to 5.52 ± 0.17%. The lowest amount of ALA (1.89 ± 0.10%) was detected in fish fed W10 with no differences in the remaining samples (ca. 2.5%), consistent with diet composition. Vasconi et al. [38] compared the fatty acid composition of the muscle from farmed seabream, collected in 2005 and 2014, reporting similar values to those of our study for C16:0, C18:1 n-9, ALA, and EPA contents. However, they attained lower amounts of C18:2 n-6 (12.47 to 16.7%) and higher contents of DHA (11.95 to 15.57%). They also observed that the replacement of fish oil by plant-based oil in aquaculture feeds led to an increase in C18:1 n-9 and C18:2 n-6 FA. In the study carried out by Pateiro et al. [39], distinct amounts of ALA (4.45%), EPA (2.25%), and DHA (5.20%) in the muscle of commercial seabream were reported. 

### 3.4. Health Lipid Indices Evaluation

The nutritional quality of food lipids is highly dependent on their fatty acid profile. Nowadays, it is well established that the consumption of saturated fatty acids promotes an increase in serum cholesterol, while polyunsaturated fatty acids, namely diets rich in n-3 PUFA, can depress total and low-density lipoprotein cholesterol levels [40]. The impact of diet on cardiovascular health can be assessed by the PUFA/SFA ratio, with values higher than 0.45 recommended to prevent cardiovascular and some chronic diseases [30]. Nevertheless, different SFAs have distinct influences on serum cholesterol. In fact, C12:0 and C14:0 are the most atherogenic agents, C16:0 is both an athero- and thrombogenic agent, whereas C18:0 is considered to be thrombogenic but neutral with respect to atherogenicity [30]. Additionally, the quality of PUFA is a feature to consider in health lipid evaluation. In fact, n-3 and n-6 PUFA compete for the same enzymes (elongases and desaturases) and have distinct effects on human health. Therefore, an appropriate n-3/n-6 ratio between 1:1 and 5:1 is recommended to prevent several chronic diseases [41]. 

In this study, fish fed W10 had the lowest values of PUFA/SFA (1.43 ± 0.05) and n-3/n-6 (1.22 ± 0.02) ratios, being statistically different from the remaining samples. Nevertheless, attending to these parameters, all the fish samples fulfill the requirements of a healthy lipid source. However, these parameters do not account for MUFA effect, particularly oleic acid which may increase the activity of low-density lipoprotein receptors, leading to a decrease in serum cholesterol [42]. For this reason, further indices are used to evaluate health lipid quality, namely the ratio between hypocholesterolemic and hypercholesterolemic fatty acids (h/H), atherogenicity index (AI), and thrombogenicity index (TI). Aiming to assess the effect of specific fatty acids in cholesterol metabolism, the ratio between hypocholesterolemic (*cis*-C18:1 and PUFA) and hypercholesterolemic (C14:0 and C16:0) fatty acids (h/H index) is frequently used. A high value of this parameter is recommended from a nutritional point of view. The AI index establishes the relation between the proatherogenic SFAs, promoters of lipid adhesion to cells of the immune and circulatory systems, and the main classes of antiatherogenic unsaturated fatty acids (UFAs), that inhibit the aggregation of plaque and decrease the levels of esterified fatty acids, cholesterol, and phospholipids, thereby preventing coronary diseases. The TI index evaluates the tendency for clot formation in the blood vessels. This is defined as the relation between the prothrombogenic (SFA) and antithrombogenic fatty acids (MUFA, n-6 and n-3 PUFA) [43]. Diets containing lipids with AI and TI lower than 1.0 and 0.5, respectively, are nutritionally recommended [44]. 

In our study, the h/H index showed a statistically significant difference between fish fed W10 (3.64 ± 0.09) and the remaining samples (ranging from 3.16 ± 0.13 to 3.36 ± 0.14). Moreover, similar values were obtained for AI (from 0.36 ± 0.01 to 0.39 ± 0.01) and for TI (from 0.29 ± 0.01 to 0.31 ± 0.02), regardless of the provided fish diet. These results highlight that fish fed diets supplemented with *P. canaliculata*, especially those fed W10, are sources of healthy lipids that might be consumed, on a regular basis, to prevent cardiovascular diseases. 

The susceptibility of a tissue to oxidation may be evaluated by the peroxidizability index (PI), highly dependent on the unsaturation degree of FA, providing information about the technological quality of the fish fillets; the higher the PI, the higher the susceptibility to oxidation [45]. In this study, PI values ranged from 115.6 ± 2.0 (W10 sample) to 130.5 ± 7.4 (Pc10), being considerably lower than those reported by Testi et al. [45], between 155 and 170, in seabream fillets. 

**Figure 4:**
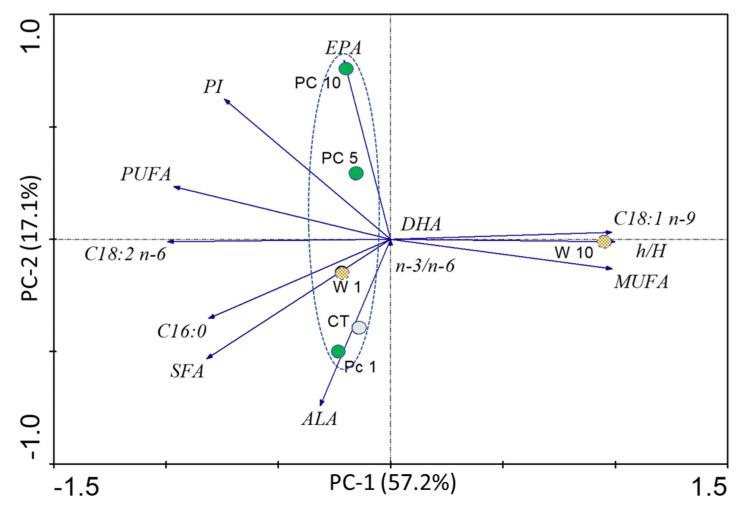
Principal component analysis (PCA) biplot of muscle samples of seabream fed with the diets under study (CT; Pc1; Pc5; Pc10; W1 and W10). The following parameters were considered: most abundant fatty acids C18:1 n-9, C18:2 n-6, and C16:0; essential fatty acids ALA, EPA, and DHA; total saturated fatty acids (SFA); total monounsaturated fatty acids (MUFA); total polyunsaturated fatty acids (PUFA); n-3 PUFA/n-6 PUFA ratio (n-3/n-6), hypocholesterolemic/hypercholesterolemic index (h/H), atherogenic index (AI), thrombogenic index (TI), and peroxidizability index (PI). PC-1 and PC-2 explain 57.2% and 17.1% of data variability, respectively.

Aiming to provide a comparative perspective of the fatty acid profile of fish fed the diets under study, a principal component analysis (PCA) was performed (Figure 4). The first principal component, PC-1, representing 57.2% of the sample variability, is mainly defined by the values of hypocholesterolemic/hypercholesterolemic index (h/H), MUFA, C18:1 n-9, C18:2 n-6, PUFA, and C16:0. The second principal component, PC-2, explains 17.1% of the sample variability and is more related with EPA and ALA values. In this figure, W10 stands out for its distinct fatty acid profile, particularly concerning the highest C18:1 n-9, MUFA, and h/H values and the lowest C18:2 n-6, PUFA, C16:0, SFA contents, and PI index.

## 4. Conclusions

This study developed a viable strategy for the use of the brown seaweed *Pelvetia canaliculata* as an ingredient in *S. aurata* diets, aiming to valorize this marine resource. In fact, fish fed a diet with 10% of algal residue (W10), that was left after sunflower oil supplementation to increase its oxidative stability, showed slightly improved values of hypocholesterolemic/hypercholesterolemic (h/H) and of peroxidizability index (PI). Nevertheless, in the remaining samples, no significant differences were observed in the proximate composition and the fatty acid profile of fish fed diets supplemented with *P. canaliculata*, in comparison with the control sample. These results support the nutritional potential of this seaweed as an ingredient in aquaculture feed. In future works, it would be interesting to carry out sensory analyses of fish fed supplemented diets, in comparison with a control sample, to evaluate the acceptance of consumers.

## Figures and Tables

**Table 1:** Fatty acid profile (% total fatty acid) of *Pelvetia canaliculata*.

Fatty Acid	Abreviation	% Total FA
Lauric acid	C12:0	0.10 ± 0.01
Tridecanoic acid	C13:0	0.07 ± 0.00
Myristic acid	C14:0	9.78 ± 0.28
Pentadecanoic acid	C15:0	0.43 ± 0.01
Palmitic acid	C16:0	13.40 ± 0.22
Heptadecanoic acid	C17:0	0.24 ± 0.01
Stearic acid	C18:0	1.54 ± 0.04
Arachidic acid	C20:0	0.34 ± 0.01
Behenic acid	C22:0	0.38 ± 0.01
Lignoceric acid	C24:0	0.42 ± 0.02
Myristoleic acid	C14:1 n-5	0.15 ± 0.01
Palmitoleic Acid	C16:1 n-7	1.65 ± 0.03
Elaidic acid	C18:1 n-9 *trans*	0.19 ± 0.01
Oleic acid	C18:1 n-9	29.59 ± 0.40
11*Z*-Octadecenoic acid	C18:1 n-7	0.33 ± 0.02
7*Z*-Octadecenoic acid	C18:1 n-11	0.16 ± 0.01
11*Z*-Eicosenoic acid	C20:1 n-9	0.13 ± 0.01
9*Z*-Eicosenoic acid	C20:1 n-11	0.03 ± 0.01
Linoleic acid	C18:2 n-6	9.95 ± 0.03
11*Z*,14*Z*-Octadecadienoic acid	C18:2 n-4	0.07 ± 0.00
γ-Linolenic acid	C18:3 n-6	1.20 ± 0.02
α-Linolenic acid (ALA)	C18:3 n-3	5.30 ± 0.13
Stearidonic acid	C18:4 n-3	1.32 ± 0.02
11*Z*, 14*Z*-Eicosadienoic acid	C20:2 n-6	0.67 ± 0.01
8*Z*, 11Z, 14*Z*-Eicosatrienoic acid	C20:3 n-6	1.73 ± 0.05
Arachidonic acid	C20:4 n-6	16.17 ± 0.19
Eicosapentaenoic acid (EPA)	C20:5 n-3	4.69 ± 0.07
	SFA	26.68 ± 0.48
	MUFA	32.23 ± 0.39
	PUFA	41.09 ± 0.29
	n-3	11.31 ± 0.19
	n-6	29.71 ± 0.26
	n-6/n-3	2.63 ± 0.05
	PUFA/SFA	1.54 ± 0.03

SFA—saturated fatty acid; MUFA—monounsaturated fatty acids; PUFA—polyunsaturated fatty acids.

**Table 2:** Fatty acid profile (% total fatty acids) of the formulated diets: CT, Pc1, Pc5, Pc10, W1, and W10.

Fatty Acid (%)	CT	Pc1	Pc5	Pc10	W1	W10
C12:0	0.13 ± 0.01 ^a^	0.15 ± 0.01 ^a b^	0.15 ± 0.01 ^ab^	0.17 ± 0.00 ^b^	0.16 ± 0.01 ^b^	0.14 ± 0.02 ^ab^
C14:0	3.46 ± 0.09 ^a^	3.84 ± 0.01 ^b^	3.99 ± 0.04 ^b^	4.23 ± 0.08 ^c^	3.94 ± 0.03 ^b^	3.92 ± 0.08 ^b^
C15:0	0.28 ± 0.00 ^a^	0.30 ± 0.00 ^b^	0.30 ± 0.00 ^b^	0.31 ± 0.01 ^c^	0.30 ± 0.00 ^b^	0.29 ± 0.01 ^b^
C16:0	17.11 ± 0.20 ^a^	17.78 ± 0.01 ^b^	17.72 ± 0.03 ^b^	17.82 ± 0.15 ^b^	17.72 ± 0.1 ^b^	15.97 ± 0.16 ^c^
C17:0	0.31 ± 0.00 ^a^	0.32 ± 0.00 ^b^	0.32 ± 0.00 ^b^	0.32 ± 0.00 ^b^	0.32 ± 0.00 ^b^	0.29 ± 0.00 ^c^
C18:0	3.77 ± 0.05 ^a^	3.69 ± 0.03 ^a^	3.73 ± 0.01 ^a^	3.75 ± 0.03 ^a^	3.69 ± 0.04 ^a^	3.54 ± 0.01 ^b^
C14:1 n-5	0.02 ± 0.00	0.02 ± 0.00	0.03 ± 0.01	0.03 ± 0.00	n.d.	n.d.
C16:1 n-7	4.70 ± 0.09 ^a^	4.96 ± 0.00 ^b^	4.92 ± 0.00 ^b^	4.99 ± 0.06 ^b^	4.98 ± 0.03 ^b^	4.93 ± 0.07 ^b^
C18:1 n-9	17.47 ± 0.01 ^ab^	17.41 ± 0.01 ^a^	17.49 ± 0.06 ^ab^	17.69 ± 0.06 ^b^	18.61 ± 0.02 ^c^	30.00 ± 0.19 ^d^
C18:1 n-7	2.29 ± 0.03 ^a^	2.25 ± 0.02 ^ab^	2.25 ± 0.01 ^b^	2.24 ± 0.00 ^b^	2.24 ± 0.01 ^b^	2.07 ± 0.01 ^c^
C20:1 n-9	0.77 ± 0.03	0.70 ± 0.00	0.70 ± 0.00	0.70 ± 0.01	0.69 ± 0.00	0.70 ± 0.01
C16:3 n-4	0.54 ± 0.01 ^a^	0.57 ± 0.00 ^b^	0.57 ± 0.00 ^b^	0.58 ± 0.01 ^b^	0.57 ± 0.01 ^b^	0.57 ± 0.01 ^b^
C16:4 n-1	0.60 ± 0.06	0.66 ± 0.02	0.61 ± 0.01	0.58 ± 0.01	0.65 ± 0.03	0.67 ± 0.02
C18:2 n-6 *trans*	1.18 ± 0.02 ^a^	1.23 ± 0.00 ^b^	1.23 ± 0.00 ^b^	1.24 ± 0.02 ^b^	1.26 ± 0.01 ^b^	1.23 ± 0.02 ^b^
C18:2 n-6 *cis*	27.83 ± 0.04 ^a^	27.55 ± 0.04 ^b^	27.11 ± 0.02 ^c^	26.35 ± 0.07 ^d^	26.77 ± 0.04 ^e^	18.12 ± 0.03 ^f^
C18:3 n-6	0.18 ± 0.01	0.19 ± 0.03	0.20 ± 0.00	n.d.	0.19 ± 0.01	0.17 ± 0.01
C18:3 n-4	0.50 ± 0.01	0.47 ± 0.01	0.47 ± 0.01	0.36 ± 0.08	0.36 ± 0.08	0.38 ± 0.09
C18:3 n-3 (ALA)	3.21 ± 0.01 ^a^	3.19 ± 0.00 ^a^	3.22 ± 0.03 ^a^	3.21 ± 0.01 ^a^	3.10 ± 0.01 ^b^	2.14 ± 0.02 ^c^
C18:4 n-3	1.07 ± 0.01 ^a^	1.07 ± 0.00 ^a^	1.09 ± 0.00 ^b^	1.13 ± 0.00 ^c^	1.07 ±0.00 ^a^	1.10 ±0.00 ^d^
C20:2 n-6	0.14 ± 0.02	0.03 ± 0.00	0.14 ± 0.02	0.02 ± 0.01	0.12 ± 0.01	0.13 ± 0.02
C20:4 n-6	0.73 ± 0.05	0.70 ± 0.00	0.89 ± 0.00	1.14 ± 0.03	0.68 ± 0.01	0.90 ± 0.05
C20:5 n-3 (EPA)	8.13 ± 0.13 ^a^	7.80 ± 0.02 ^b^	7.74 ± 0.01 ^b^	7.89 ± 0.13 ^b^	7.66 ± 0.07 ^b^	7.75 ± 0.07 ^b^
C22:5 n-3	0.78 ± 0.03	0.71 ± 0.00	0.72 ± 0.01	0.72 ± 0.01	0.70 ± 0.01	0.71 ± 0.01
C22:6 n-3 (DHA)	4.81 ± 0.21 ^a^	4.38 ± 0.02 ^b^	4.41 ± 0.01 ^b^	4.44 ± 0.09 ^b^	4.27 ± 0.04 ^b^	4.30 ± 0.05 ^b^
SFA	25.06 ± 0.26 ^a^	26.09 ± 0.03 ^b^	26.22 ± 0.07 ^b^	26.60 ± 0.23 ^b^	26.13 ± 0.14 ^b^	24.16 ± 0.27 ^c^
MUFA	25.25 ± 0.05 ^a^	25.35 ± 0.01 ^a^	25.38 ± 0.06 ^a^	25.66 ± 0.03 ^b^	26.53 ± 0.02 ^c^	37.70 ± 0.14 ^d^
PUFA	49.70 ± 0.31 ^a^	48.56 ± 0.04 ^b^	48.40 ± 0.02 ^b^	47.75 ± 0.23 ^c^	47.34 ± 0.15 ^c^	38.15 ± 0.19 ^d^
n-3	18.00 ± 0.36 ^a^	17.15 ± 0.03 ^bc^	17.18 ± 0.04 ^bc^	17.40 ± 0.24 ^b^	16.80 ± 0.12 ^c^	16.00 ± 0.15 ^d^
n-6	30.06 ± 0.04 ^a^	29.70 ± 0.07 ^b^	29.57 ± 0.04 ^b^	28.82 ± 0.06 ^c^	28.95 ± 0.06 ^c^	20.54 ± 0.06 ^d^
n-3/n-6	0.60 ± 0.03 ^ab^	0.58 ± 0.01 ^c^	0.58 ± 0.01 ^ac^	0.60 ± 0.02 ^b^	0.58 ± 0.01 ^c^	0.78 ± 0.01 ^d^

For each parameter a distinct letter means significative differences (*p* ≤ 0.050) between samples in the same row. DHA—docosahexaenoic acid. n.d.—not detected.

**Table 3:** Fatty acid profile (% total fatty acids) of muscle of *S. aurata* fed with the formulated diets: CT, Pc1, Pc5, Pc10, W1, and W10.

Fatty Acid (%)	CT	Pc 1	Pc 5	Pc 10	W1	W10
C14:0	3.09 ± 0.08 ^ab^	3.13 ± 0.06 ^ab^	3.16 ± 0.13 ^ab^	3.22 ± 0.13 ^a^	3.03 ± 0.06 ^b^	3.11 ± 0.04 ^ab^
C15:0	0.26 ± 0.03 ^ab^	0.26 ± 0.01 ^a^	0.26 ± 0.01 ^a^	0.22 ± 0.12 ^ab^	0.24 ± 0.00 ^b^	0.26 ± 0.01 ^a^
C16:0	16.32 ± 0.41 ^ab^	16.57 ± 0.71 ^a^	16.01 ± 0.50 ^ab^	16.00 ± 0.60 ^ab^	16.78 ± 0.27 ^a^	15.46 ± 0.38 ^b^
C17:0	0.25 ± 0.06 ^a^	0.20 ± 0.02 ^a^	0.36 ± 0.01 ^b^	0.20 ± 0.02 ^a^	0.22 ± 0.07 ^a^	0.19 ± 0.04 ^a^
C18:0	4.46 ± 0.20 ^ab^	4.37 ± 0.22 ^ab^	4.31 ± 0.11 ^ab^	4.28 ± 0.13 ^a^	4.57 ± 0.09 ^b^	3.96 ± 0.13 ^c^
C21:0	0.33 ± 0.04	0.32 ± 0.05	0.37 ± 0.05	0.29 ± 0.05	0.32 ± 0.03	0.35 ± 0.06
C16:1 n-7	5.12 ± 0.13	5.17 ± 0.10	5.21 ± 0.14	5.10 ± 0.17	5.08 ± 0.13	5.18 ± 0.07
C18:1 n-9 *trans*	0.15 ± 0.04	0.23 ± 0.07	0.18 ± 0.05	0.15 ± 0.03	0.16 ± 0.06	0.15 ± 0.03
C18:1 n-9 *cis*	23.52 ± 0.73 ^a^	23.11 ± 0.85 ^a^	24.13 ± 1.82 ^a^	23.51 ± 2.19 ^a^	25.27 ± 1.62 ^a^	35.37 ± 0.31 ^b^
C18:1 n-7	3.54 ± 0.11	3.55 ± 0.19	3.40 ± 0.02	3.72 ± 0.08	n. d	n. d
C18:1 n-11	0.67 ± 0.02 ^a^	0.66 ± 0.06 ^a^	0.47 ± 0.01 ^b^	0.61 ± 0.07 ^a^	0.52 ± 0.09 ^b^	0.68 ± 0.03 ^a^
C20:1 n-9	0.93 ± 0.10 ^a^	0.82 ± 0.06 ^ab^	0.82 ± 0.09 ^ab^	0.81 ± 0.04 ^ab^	0.75 ± 0.06 ^b^	0.90 ± 0.03 ^a^
C20:1 n-11	0.81 ± 0.07	0.80 ± 0.07	0.78 ± 0.03	0.79 ± 0.04	0.76 ± 0.04	0.78 ± 0.04
C24:1 n-9	0.57 ± 0.07 ^ab^	0.58 ± 0.07 ^ab^	0.60 ± 0.06 ^a^	0.62 ± 0.05 ^a^	0.48 ± 0.04 ^b^	0.55 ± 0.09 ^ab^
C18:2 n-6	22.63 ± 0.39 ^a^	23.03 ± 0.66 ^a^	23.29 ± 0.90 ^a^	23.25 ± 0.69 ^a^	23.67 ± 0.76 ^a^	16.77 ± 0.28 ^b^
C18:3 n-6	0.38 ± 0.05 ^ab^	0.46 ± 0.10 ^a^	0.50 ± 0.07 ^a^	0.42 ± 0.10 ^ab^	0.45 ± 0.07 ^ab^	0.32 ± 0.04 ^b^
C18:3 n-3 (ALA)	2.52 ± 0.02 ^a^	2.53 ± 0.14 ^a^	2.46 ± 0.06 ^a^	2.48 ± 0.11 ^a^	2.46 ± 0.06 ^a^	1.89 ± 0.10 ^b^
C18:4 n-3	0.17 ± 0.04	0.21 ± 0.06	0.18 ± 0.02	0.17 ± 0.07	0.17 ± 0.06	0.17 ± 0.03
C20:2 n-6	0.40 ± 0.05 ^ab^	0.37 ± 0.06 ^ab^	0.45 ± 0.09 ^a^	0.41 ± 0.03 ^ab^	0.37 ± 0.03 ^ab^	0.34 ± 0.04 ^b^
C20:3 n-6	0.32 ± 0.03 ^a^	0.35 ± 0.04 ^ab^	0.42 ± 0.06 ^b^	0.39 ± 0.04 ^b^	0.37 ± 0.03 ^ab^	0.32 ± 0.02 ^a^
C20:4 n-6	0.61 ± 0.09 ^a^	0.61 ± 0.09 ^a^	0.80 ± 0.06 ^b^	0.76 ± 0.09 ^b^	0.57 ± 0.08 ^a^	0.56 ± 0.03 ^a^
C20:5 n-3 (EPA)	5.25 ± 0.21	5.30 ± 0.18	5.43 ± 0.17	5.52 ± 0.17	5.40 ± 0.13	5.31 ± 0.10
C22:5 n-3	1.91 ± 0.08	1.79 ± 0.10	1.91 ± 0.21	1.94 ± 0.10	1.93 ± 0.10	1.95 ± 0.03
C22:6 n-3 (DHA)	5.79 ± 0.53 ^ab^	5.74 ± 0.35 ^ab^	6.40 ± 0.30 ^a^	6.36 ± 0.73 ^a^	5.52 ± 0.13 ^b^	5.73 ± 0.20 ^ab^
SFA	24.71 ± 0.52 ^a^	24.85 ± 0.80 ^a^	24.47 ± 0.60 ^a^	24.21 ± 0.58 ^ab^	25.16 ± 0.27 ^a^	23.33 ± 0.43 ^b^
MUFA	35.30 ± 0.62 ^a^	34.82 ± 0.58 ^ab^	33.86 ± 0.87 ^b^	34.08 ± 0.85 ^ab^	33.91 ± 0.86 ^b^	43.31 ± 0.27 ^c^
PUFA	39.99 ± 0.84 ^a^	40.34 ± 1.34 ^a^	41.66 ± 0.93 ^a^	41.71 ± 1.30 ^a^	40.93 ± 1.02 ^a^	33.37 ± 0.59 ^b^
n-3	15.63 ± 0.76 ^ab^	15.57 ± 0.61 ^ab^	16.34 ± 0.48 ^a^	16.48 ± 0.89 ^a^	15.50 ± 0.25 ^ab^	15.05 ± 0.27 ^b^
n-6	24.3 ± 0.41 ^a^	24.76 ± 0.88 ^a^	25.32 ± 0.85 ^a^	25.23 ± 0.74 ^a^	25.44 ± 0.82 ^a^	18.31 ± 0.36 ^b^
DFA	79.74 ± 0.39 ^ab^	79.52 ± 0.69 ^a^	79.83 ± 0.57 ^ab^	80.07 ± 0.70 ^ab^	79.41 ± 0.30 ^a^	80.63 ± 0.38 ^b^
EFA	25.76 ± 0.35 ^a^	26.16 ± 0.84 ^a^	26.56 ± 0.94 ^a^	26.49 ± 0.70 ^a^	26.71 ± 0.80 ^a^	19.22 ± 0.35 ^b^
UFA/SFA	3.05 ± 0.09 ^a^	3.03 ± 0.13 ^a^	3.09 ± 0.10 ^a^	3.13 ± 0.10 ^ab^	2.97 ± 0.04 ^a^	3.29 ± 0.08 ^b^
PUFA/SFA	1.62 ± 0.06 ^a^	1.63 ± 0.11 ^a^	1.70 ± 0.07 ^a^	1.72 ± 0.09 ^a^	1.63 ± 0.05 ^a^	1.43 ± 0.05 ^b^
n-3/n-6	1.56 ± 0.08 ^a^	1.59 ± 0.05 ^a^	1.55 ± 0.07 ^a^	1.53 ± 0.09 ^a^	1.64 ± 0.04 ^a^	1.22 ± 0.02 ^b^
h/H	3.21 ± 0.08 ^a^	3.16 ± 0.13 ^a^	3.36 ± 0.14 ^a^	3.22 ± 0.16 ^a^	3.27 ± 0.13 ^a^	3.64 ± 0.09 ^b^
AI	0.38 ± 0.00 ^ab^	0.39 ± 0.01 ^a^	0.38 ± 0.01 ^ab^	0.38 ± 0.02 ^ab^	0.39 ± 0.01 ^a^	0.36 ± 0.01 ^b^
TI	0.31 ± 0.01	0.31 ± 0.02	0.29 ± 0.01	0.29 ± 0.02	0.31 ± 0.01	0.29 ± 0.01
PI	122.8 ± 5.8 ^ab^	122.7 ± 4.5 ^ab^	130.1 ± 3.0 ^a^	130.5 ± 7.4 ^a^	122.6 ± 2.6 ^ab^	115.6 ± 2.0 ^b^

For each parameter, a distinct letter means significative differences (*p* ≤ 0.050) between samples in the same row. h/H—hypocholesterolemic/hypercholesterolemic index; AI—atherogenic index; TI—thrombogenic; PI—peroxidizability index. n.d.—not detected.

## Data Availability

Data are available on request to the authors.
